# Virtues, ecological momentary assessment/intervention and smartphone technology

**DOI:** 10.3389/fpsyg.2015.00481

**Published:** 2015-05-06

**Authors:** Jason D. Runyan, Ellen G. Steinke

**Affiliations:** Psychology Department, Indiana Wesleyan UniversityMarion, IN, USA

**Keywords:** experience sampling, mindfulness, self-awareness, habits, automaticity, character traits, virtues, dispositions

## Abstract

Virtues, broadly understood as stable and robust dispositions for certain responses across morally relevant situations, have been a growing topic of interest in psychology. A central topic of discussion has been whether studies showing that situations can strongly influence our responses provide evidence against the existence of virtues (as a kind of stable and robust disposition). In this review, we examine reasons for thinking that the prevailing methods for examining situational influences are limited in their ability to test dispositional stability and robustness; or, then, whether virtues exist. We make the case that these limitations can be addressed by aggregating repeated, cross-situational assessments of environmental, psychological and physiological variables within everyday life—a form of assessment often called ecological momentary assessment (EMA, or experience sampling). We, then, examine how advances in smartphone application (app) technology, and their mass adoption, make these mobile devices an unprecedented vehicle for EMA and, thus, the psychological study of virtue. We, additionally, examine how smartphones might be used for virtue development by promoting changes in thought and behavior within daily life; a technique often called ecological momentary intervention (EMI). While EMA/I have become widely employed since the 1980s for the purposes of understanding and promoting change amongst clinical populations, few EMA/I studies have been devoted to understanding or promoting virtues within non-clinical populations. Further, most EMA/I studies have relied on journaling, PDAs, phone calls and/or text messaging systems. We explore how smartphone app technology provides a means of making EMA a more robust psychological method, EMI a more robust way of promoting positive change, and, as a result, opens up new possibilities for studying and promoting virtues.

## Introduction

Over the past 15 years, virtues have received increased attention in the psychological sciences. This has, in large part, been a result of the positive psychology movement (Seligman and Csikszentmihalyi, 2000; Seligman et al., 2005). Positive psychology is a subfield of psychological science devoted to a deliberate attentiveness to human flourishing and its promotion. And virtues have been a central focus of positive psychology (Seligman et al., 2005; Kristjánsson, 2013; Worthington et al., 2014).

This increased attention to virtues, and positive psychology in general, has not gone without criticism (e.g., Gable et al., 2005; Held, 2005; Sundararajan, 2005; Kristjánsson, 2010, 2013; McNulty and Fincham, 2012). In this review, our goal is not to defend positive psychology. We, however, propose that there is virtue in the psychological study of virtues. We, further, propose that smartphone technology opens up a new means of studying, and possibly promoting, virtue. In making our case, we discuss:

- philosophical and psychological work concerning virtue stability (persistence over time) and robustness (cross-situational persistence);
- smartphone application (app) advances pertaining to the measurement of human (and physiological) activity and experience within daily life; and
- the use of mobile app technology for ecological momentary assessment and intervention as it pertains to the study of virtues.

## Virtues and situational studies: questions of stability and robustness

Dating at least as far back as Aristotle's analytic treatment of virtues, there has been a long history of understanding virtues as a kind of disposition (*hexis*). This Aristotelian conception is often referred to as the traditional conception (cf. Timpe and Boyd, 2014); and, following a marked decline in interest, virtues understood along Aristotelian lines have received renewed attention in moral philosophy. This renewed attention is in large part a result of the influence of works by Anscombe (1958), Foot (1978), and MacIntyre (1984).

According to a traditional Aristotelian conception, virtues are human excellences understood as a *subclass* of psychological disposition, or that is, ability to behave or think in certain ways, or have certain psychological responses, across relevant situations (Aristotle, 2000 I.13; Aristotle *EE* II.1218-20). Expressing virtues is constitutive of a flourishing (*eudaimonic*) life, or, that is, a deeply fulfilling, well-lived life of growth (cf. Aristotle *EE* I.7-II.1; Fowers, 2012). While a person can be virtuous without flourishing, and even while suffering, a person cannot flourish without being virtuous^1^. In the way a good ax cuts wood well, a flourishing person expresses virtues (Aristotle *EE* II.1213-34). And virtues are expressed by actively doing or thinking certain things, or by feeling certain emotions or having certain passive responses (e.g., refraining from certain behavior), in morally relevant situations (cf. Jayawickreme and Chemero, 2008).

Virtues, understood as a subclass of disposition, have been recognized as having three important characteristics (Timpe and Boyd, 2014). First, they are relatively *stable*. Virtues tend to persist over some period of time. Thus, we generally cannot be sure whether someone has a virtue until they have expressed it on multiple occasions. Similarly, we generally cannot be sure whether someone has a virtue until they have expressed it under various virtue-relevant situations. And this relates to the second characteristic of virtues. Virtues are relatively *robust* in the sense that they are consistently expressed across a range of situations. Third, virtues are *interconnected* in the sense that having one virtue increases the probability of having others (cf. Watson, 1984; MacIntyre, 1999; Annas, 2011).

In modern psychology, Allport provided an early treatment that supported the conception of virtues as a kind of relatively stable, robust and interconnected disposition or trait (cf. Allport, 1960)^2^. However, more recently, a number of moral psychologists have argued that there are good experimental grounds for thinking virtues do not actually exist (cf. Nahmias, 2010). The argument is that virtues are no more than unmaterialized ideals. The grounds for this argument come from studies indicating that, to a significant degree, a person's situation can influence their conduct and thought without them being aware of it (Hunt, 1965; Mischel, 1968; Ross and Nisbett, 1991; Doris, 1998, 2002; Harman, 1999). In one of the most well-known of these studies—Zimbardo's Stanford Prison Experiment—college students began exhibiting guard-like or inmate-like behavior after only a few days of taking on the role of either a guard or a prisoner in a mock prison (Haney et al., 1973). In another well-known experiment, Milgram (1963) found that a majority of participants would administer what they thought to be a potentially lethal shock to individuals they had never met if ordered to by an experimenter as part of what was presented to them as a scientific study (see also Hartshorne and May, 1928; Asch, 1951; Isen and Levin, 1972). Additionally, over the past 20 years, a wealth of studies have indicated that priming individuals by having them, for example, read words with either prosocial or antisocial connotations, or handle certain kinds of objects (e.g., smooth, rough, heavy, hot, cold), can influence their subsequent behavior and judgments without them realizing it (e.g., Williams and Bargh, 2008; Ackerman et al., 2010; Bargh and Shalev, 2012).

The kinds of situational studies mentioned above have been thought to provide evidence against the existence of virtues as relatively stable and robust dispositions; that is, as dispositions consistently expressed across relevant situations over a period of time (Harman, 1999; Doris, 2002). However, while drawing attention to the extent and ways situations can influence individuals, to think these studies provide evidence against the existence of virtues, conceived of as a type of stable and robust disposition, is to infer too much. As Croom (2014) and others (e.g., Alzola, 2008) have recently pointed out, thinking these studies provide evidence against the existence of virtues results from: oversimplifying an Aristotelian conception of virtues as perfectly stable and robust dispositions; and/or drawing unwarranted conclusions from these studies.

First, as Anscombe (1958, p. 14) has pointed out, regardless of the virtues possessed by the average person, there may be a complete set of virtues each of which is possessed by some people. Virtues may be rare without being nonexistent; and, granted they exist, they are likely rare. Thus, when studying whether virtues exist, it is necessary to distinguish candidates for having a certain stable and robust disposition from other individuals in order to examine whether relevant situational influences have similar effects on both groups. So it is necessary to examine exemplars (e.g., Colby and Damon, 1992, 1999; Dunlop and Walker, 2013). Situational factors may not influence exemplars in the way they do the average person. Exemplars may express a virtue despite situational influences that make it difficult to do so. This, however, has not been tested in the situational studies purported to call the existence of virtues in question. As a result, the situational effects observed in these studies should not be generalized to the entire human population. Even if such situational effects are found in the majority, this does not provide evidence that virtues do not exist.

Second—and in keeping with the above—the situational studies purported to provide evidence that virtues are nonexistent do not even provide evidence that virtues are not possessed by a subgroup within the samples studied (cf. Miller, 2013). Thus, at most, these studies indicate that certain virtues are rare. For instance, in Darley and Batson's (1973) “Good Samaritan” study, only 10% of seminarians that participated in the study helped someone who appeared to need medical attention as they rushed to give a talk for which they were very late. While these observations might be taken to indicate that 90% of the seminarians lack a certain virtue, it clearly should not be taken to indicate that all of them do.

Third, many of the studies thought to call into question the existence of virtues have used undergraduate students who may still be developing in ways relevant to the development of virtues. It has been observed that the prefrontal cortex, and its connectivity to other regions, typically continues to develop up to late- or post-adolescence. This development is inversely correlated with novelty seeking (e.g., Pfefferbaum et al., 1994; Reiss and Havercamp, 1996; Sowell et al., 1999, 2003; Gogtay et al., 2004; Kelley et al., 2004; Segalowitz and Davies, 2004; Somerville et al., 2010, 2011) and impulsivity (Shannon et al., 2011), which are, in turn, likely to be inversely correlated with possessing *stable* and *robust* psychological dispositions, including virtues. Thus, many of these studies are performed using samples that tend toward not having yet developed certain, stable psychological dispositions.

Fourth, to provide evidence that virtues do not exist, it would need to be shown that engaging in practices thought to contribute to virtue development does not mitigate situational influences on an individual's responses. However, none of the situational studies thought to bring the existence of virtues in question tests this.

Fifth, a substantial amount of evidence indicates that cross-situational consistencies in responses characteristic of relatively stable and robust dispositions—such as virtues—are often missed without the aggregation of repeated, cross-situational measurements (Dlugokinski and Firestone, 1973, 1974; Staub, 1974; Epstein, 1979, 1983; Rushton, 1980, 1984; Rushton et al., 1981, 1983; Fleeson, 2001; Furr, 2009). The reason is there are multiple responses characteristic of these kinds of dispositions and there is some degree of variability in their expression as a result of interfering factors (Fleeson and Noftle, 2008; Miller, 2013). Thus, using one or two situational tests to examine whether an individual possesses a disposition, or virtue, is an insufficient and unreliable approach to testing dispositional stability and robustness.

In sum, situational studies thought to provide evidence against the existence of virtues are not optimally designed to test whether virtues exist, and, thus, should not be taken to indicate they do not. There is, on the other hand, accumulating evidence that at least some individuals do possess virtues.

First, studies that aggregate repeated, cross-situational measurements of responses provide evidence that at least some individuals express relatively stable and robust psychological dispositions (Ozer, 1986; Fleeson, 2001, 2004; McNiel and Fleeson, 2006; John et al., 2008; Donnellan and Lucas, 2009), even if they are not impervious to situational influences (Fleeson, 2007; Fleeson and Noftle, 2008; Bleidorn, 2009). It is likely that some of these dispositions contribute to psychological health and growth and so, in keeping with a broadly conceived Aristotelian tradition, are virtues.

Second, it has been observed that at least some psychological states tend to fluctuate around a “set point” (e.g., Suh et al., 1996; Diener and Lucas, 1999; Diener et al., 2006; Keltner, 2009). Since a psychological state can be the expression of a disposition, this observation provides further evidence that relatively stable and robust psychological dispositions exist. And, again, it is likely that some of these dispositions contribute to psychological health and growth; and so—again in keeping with an Aristotelian tradition—are virtues.

Third, virtues can be possessed in degrees and the degree to which a virtue is possessed can be used to predict patterns of responses, such as the frequency of virtue-relevant responses across relevant situations (cf. Miller, 2013). At the same time, as with all dispositions, there are factors that can interfere with the expression of virtues. Thus, some variability in virtue expression across relevant situations is to be expected, and this variability can be used to measure degree of virtue possession (Miller, 2013).

Finally, as we will see in what follows, there is growing psychological and neurophysiological evidence suggesting that training can promote the development of relatively stable and robust psychological dispositions which contribute to flourishing; or, that is, to virtue development understood along Aristotelian lines.

In this review we make the case that advances in smartphone technology open up a new approach to the psychological study of relatively stable and robust dispositions. We also make the case that putting this technology to use in this capacity promises to add to mounting psychological and neurophysiological evidence that certain individuals possess virtues understood along Aristotelian lines. Having stated this, we should be careful not to undervalue longstanding, everyday evidence that, throughout history, certain individuals have consistently expressed virtues in spite of strong situational influences to the contrary (see Colby and Damon, 1992). For instance, steadfast individuals who hid Jews in Nazi Germany, or undermined the Nazi regime in other ways, have been well documented and readily come to mind (e.g., see Marsh, 2014). We should, instead, seek to learn from such exemplars. Further, we should be careful not to undervalue the fact virtues have remained a useful, relevant and commonplace construct for thousands of years.

Keeping the above in mind, we propose the integration of something ancient and something cutting-edge: the study of virtues and the use of smartphone app technology. We propose that recent advancements in smartphone app technology, and the mass adoption of this technology, opens up a new means of examining and developing virtues through ecological momentary assessment (EMA) and ecological momentary intervention (EMI), respectively. In the remainder of this review, we, first, introduce EMA and discuss how smartphone technology provides a vehicle for making EMA a robust psychological method. We, second, examine how smartphone EMA studies promise to add to our knowledge of virtues; and, in particular, virtue stability and robustness. We, then, introduce EMI and examine how smartphone technology provides a vehicle for making EMI a widespread and effective way of promoting positive change. Finally, we discuss the promise smartphone EMI holds as an effective means of promoting virtue development.

## EMA and smartphone apps

Quantitative psychological studies have, traditionally, relied heavily on surveys and laboratory experiments. Both approaches have well-known and long-endured limitations. Surveys require people to make retrospective and often generalized judgments, which tend to be affected by memory limitations and recall biases (cf. Schwarz, 2007). Laboratory experiments do not occur within the context of a person's daily life; and context can influence a person's states and responses (cf. Hammond et al., 1998; Wilhelm et al., 2011), which raises questions about ecological validity (Shiffman et al., 2008). Additionally, since neither surveys nor laboratory experiments involve repeated, cross-situational sampling, both approaches fail to detect intrasubject variability within the context of an individual's everyday life (cf. Hammaker, 2012). This is particularly relevant with respect to studying virtues since, as observed in the previous section, the extent to which an individual's states and responses vary across situations—as well as individual differences in this regard—are crucial to measure when examining virtue possession.

Though earlier analogs existed, ecological momentary assessment (EMA, also referred to as experience sampling or ambulatory assessment) developed in the 1980s as a way of addressing the limitations of traditional quantitative methods in psychological science (Csikszentmihalyi and Larson, 1987; Stone and Shiffman, 1994; Shiffman et al., 2008). In particular, it was developed as a form of assessment that allowed repeated sampling within the various situations of daily life. In EMA, individuals are prompted, at fixed or random times, to respond to questions about what they are presently doing and/or experiencing (or what they have done and/or experienced in the recent past), repeatedly, throughout a period of time within the course of their daily affairs. EMA has been implemented in a number of ways, including through the use of stopwatches and paper-and-pencil diaries, PDAs (personal digital assistants; e.g., PalmPilots), phone calls and text messages. However, progress in smartphone technology has, recently, opened up a new mode of EMA.

With the release of the iPhone OS2 operating system in 2008, smartphones that could run third-party applications, or “apps,” began being used in the daily activities of millions of people. By 2009, with the release of the iPhone OS3 operating system, millions began carrying devices that could run multiple apps continuously in the background; and some of these apps could run without an internet connection. Today, a number of companies make smartphones with this capability and approximately 1.91 billion people carry these devices (eMarketer, 2015). It is projected that by 2018 this number will increase to 2.56 billion.

In more economically “developed” countries, the near ubiquitous use of smartphone apps makes EMA practical for widespread use. For the first time, EMA can be conducted in a robust and dynamic way by using a tool that is already a part of daily life for a large percentage of the population (Raento et al., 2009). The widespread use of smartphones opens up a means of collecting psychological data within the moments of daily life along with data collected through various types of sensors (e.g., global positioning systems (GPS), microphones, cameras, activity monitors, heart rate monitors). And, unlike with other modes of EMA, participants need not be trained to use a new device. Additionally, whereas using PDAs for EMA requires a certain amount of programming expertise, flexible smartphone app-based EMA systems are being developed and distributed that allow researchers to create their own EMA designs through a user-friendly web interface that requires no programming expertise (for a list see Conner, 2014; Konrath, 2015; also see Table 1)^3^. Further, smartphone app-based EMA systems have been created that automatically enter data into datasets as the data streams in from participants' smartphones. This increases the practicality of handling the relatively large amounts of data collected in EMA studies, which can easily approach ten thousand items of data.

**Table 1:** **EMA peer-reviewed psychological studies using smartphone apps**.

**References**	**Assessment target**	**App**	**Sensors**	**Tailoring**
Burns et al., 2011^a^	Depressive symptoms	Custom built	x	x
Palmier-Claus et al., 2012	Psychotic symptoms	Custom built		
Bossman et al., 2013	Affect and physical activity	Movisens	x	
von Haaren et al., 2013	Affect and physical activity	Movisens	x	
Walter et al., 2013	Mood and physical activity	Movisens		
Runyan et al., 2013	Time-management	iHabit		
Kirk et al., 2013	Illicit drug use	eMOCHA		
MacKerron and Mourato, 2013	Happiness	Mappiness		
Dunton et al., 2013^b^	Affect and physical activity amongst children	Custom built	x	
Watkins et al., 2014	Urges to smoke and location during attempts to quit smoking	Custom built	x	
Khor et al., 2014	Coping, behavior and emotion problems amongst adolescents with high-functioning autism	Mobiletype		
Garcia-Palacios et al., 2014	Chronic pain in fibromyalgia	Custom built		
Adams et al., 2014	Stress	SESAME		
Randall et al., 2014	Emotion, regulation strategies and music listening	MuPsych		
Gonzalez and Dulin, 2015	Alcohol use disorders	LBMI-A		
Ottaviani et al., 2015	Mind-wandering and cognitive rigidity	SurveyPocket and KoBo	x	
Bleidorn and Denissen, 2015	Virtues	Movisens		

*These studies were located by searching Pubmed.com, and publications from peer-reviewed psychology journals listed in Scholar.google.com, up to February 27th, 2015 (search terms: “ecological momentary assessment” and “smartphone”)*.

a *In the Burns et al. (2011) study, depressive symptoms were assessed in order to predict mood for the purposes of tailoring when coping strategies were delivered. Thus, this was an EMA/I study*.

b *Dunton et al. (2013) used custom software downloaded onto a mobile phone rather than a standard smartphone app*.

In addition to making EMA more practical for widespread use, smartphone app-based EMA systems have been designed to ensure that participants respond to questions “in the moment,” or, that is, soon after being alerted to do so. Some systems time stamp responses so that the time lapse between when a person is notified to answer a question and when they answer it can be calculated. Some systems also allow researchers to give participants a limited time window to respond to questions after being notified. These features help ensure that individuals are not merely giving a convenience sampling, which has been an issue with other modes of EMA (e.g., Stone et al., 2002).

As shown in Table 1, since 2011, there have been a number of psychological EMA studies conducted using smartphone apps. Several of these involved acquiring data through environmental, activity and/or physiological sensors together with self-report. One has involved tailoring assessments to the individual, and their moment-to-moment experiences, in response to their location and activity level.

To date, most app-based EMA studies have been conducted on clinical populations and many have been pilot studies. There have, however, been a number of non app-based EMA studies that have examined momentary dispositional expressions (see Table 2). To our knowledge, only one EMA study (published after this manuscript was under review) has systematically focused on assessing virtues using momentary responses (see Bleidorn and Denissen, 2015). Nevertheless, EMA provides a means of repeatedly measuring an individual's states, experiences and responses, as well as the extent to which these vary, using multiple measures throughout the moments and situations of everyday life. And, as we saw in the previous section, repeated, cross-situational sampling using an aggregate of measures is crucial for the psychological study of virtues; and, in particular, for testing dispositional stability and robustness. Further, as Wichers (2014) has recently observed, measuring moment-to-moment states and events can provide insight concerning patterns contributing to the development of enduring unhealthy or healthy mental conditions. Thus, app-based EMA—as a means of measuring moment-to-moment states and events—provides an unprecedented vehicle for studying dispositions, including those that contribute to psychological well-being; i.e., virtues.

**Table 2:** **EMA peer-reviewed psychological studies targeting momentary dispositional expression within non-clinical populations**.

**Study**	**Target**	**EMA mode**
Marco and Suls, 1993	Neuroticism	Paper and pencil
Stone et al., 1998; Schwartz et al., 1999; Roesch et al., 2010	Coping	PDA, PDA, Internet daily diary, respectively
Räikkönen et al., 1999; Vella et al., 2012; Demarble et al., 2014	Hostility	PDA and Monitoring devices for ECG and blood pressure
D'Antono et al., 2001	Agreeableness	Paper and pencil
Brown and Ryan, 2003; Levesque and Brown, 2007	Mindfulness	Paper and pencil
Conner and Barrett, 2005	Implicit self-attitudes and negative feeling states	PDA
Kane et al., 2007	Working memory capacity and mind-wandering	PDA
Moberly and Watkins, 2008; Huffziger et al., 2013	Ruminative self-focus	Paper and pencil, PDA, respectively
Conway et al., 2009	Empathy, altruism, helping behavior and affect	PDA
Burt and Donnellan, 2010	Antisocial behavior and acting-out	PDA
Minbashian et al., 2010	Conscientiousness	PDA
Fay and Sonnentag, 2012	Trait affect and proactive behavior	PDA
Bruehl et al., 2012	Anger and chronic pain intensity	PDA
Schwerdtfeger and Scheel, 2012	Self-esteem and cardiac vagal tone	PDA; Monitoring devices for ECG and bodily movement
Hofmann et al., 2012; Lopez et al., 2014	Self-control	PDA
Edmondson et al., 2013	Anxiety and anger	PDA
%Lopez et al., 2014	Self-control	PDA
Silvia et al., 2014	Creativity	Cell phone delivered surveys
aan het Rot et al., 2015	Impulsivity, quarrelsomeness and agreeableness	Paper and pencil

*These studies were located by searching Pubmed.com, and publications from peer-reviewed journals listed in Scholar.google.com, up to February 27th, 2015 (search terms: “ecological momentary assessment” and “virtue,” or “trait,” or “disposition”). Only studies targeting momentary dispositional expressions in non-clinical populations are included*.

## Virtues and app-based EMA

Traditionally, in Western thought, *wisdom*, *justice*, *temperance*, and *courage* have been thought of as “cardinal” (derived from the Latin “cardo” meaning hinge), or principal, virtues (e.g., Wisdom of Solomon 8:7; Plato, 380 BC/1991; Ambrose et al., (377 AD/1961)). Aristotle, however, influentially extended this list and understood virtues to be optimal points between deficiencies and excesses (cf. Aristotle *EE*; see Table 3). Additionally, within Christian theology, *faith*, *hope*, and *love* (or *charity*) have traditionally been upheld as key “theological virtues” (e.g., 1 Corinthians 13:13; Aquinas, 1274/1948).

**Table 3:** **Aristotle's list of virtues**.

**Virtue (Mean)**	**Deficiency**	**Excess**
Courage	Cowardice	Foolhardiness
Temperance	Insensibility	Intemperance
Liberality (Generosity in small matters)	Illiberality	Prodigality
Magnificence (Generosity in large matters)	Shabbiness	Extravagance
Self-worth	Diffidence	Vanity
Dignity	Servility	Churlishness
Gentleness	Impassivity	Irascibility
Candor	Dissembling	Boastfulness
Justice	Loss	Gain
Friendliness	Surliness	Flattery
Modesty	Shamelessness	Shyness
Righteous indignation	Malicious enjoyment	Envy
Wisdom	Naivety	Cunning
Hardiness	Softness	Toughness

*Aristotle understood virtues to be means between the vices of excess and deficiency. This table lists Aristotle's virtues along with their corresponding excess and deficiency (adapted from Kenny's (2011) translation of Eudemian Ethics; note: Aristotle develops a slightly different list in the Nicomachean Ethics)*.

Recently, the case has been made that six “overarching” characteristics are widely upheld as virtues across most cultures (Peterson and Seligman, 2004; Dahlsgaard et al., 2005; Seligman et al., 2005; but see Shryack et al., 2010). These are: *wisdom*, *courage*, *humanity*, *justice*, *temperance*, and *transcendence* (see Table 4). And there has been some indication that rankings of these characteristics strongly correlate across many countries (*n* = 54) and, to some extent, transcend ethnic, cultural and religious differences (Park et al., 2006; but see van Oudenhoven et al., 2012).

**Table 4:** **Peterson and Seligman's (2004) “Virtues in Action” classification of virtues**.

**Wisdom**	**Creativity, Curiosity, Open-mindedness, Love of learning,**
	**Perspective**
Courage	Authenticity, Bravery, Persistence, Zest
Humanity	Kindness, Love, Social intelligence
Justice	Fairness, Leadership, Teamwork
Temperance	Forgiveness, Modesty, Prudence, Self-regulation
Transcendence	Gratitude, Hope, Humor, Religiousness

*This table has been adapted from Seligman et al. (2005), which contains a description of the various virtue subtypes*.

However, rather than understanding virtues as a prescribed set of characteristics, following the broadly conceived Aristotelian conception we outlined earlier, we understand virtues to be a kind of relatively stable and robust psychological disposition the expression of which contributes to a fulfilling, well-lived life of growth; or, that is, to a flourishing life. Whatever else a fully flourishing life may involve, such a life involves psychological growth, psychological (*eudaimonic*) well-being and physical health (cf. Ryan and Deci, 2001; Keyes, 2007; Ryff and Singer, 2008; Deci and Ryan, 2008a)^4^. In this case, since psychological growth, psychological well-being and physical health are measurable, which dispositions contribute to a flourishing life, and, thus, should be included in a list of virtues, can be empirically studied. What should be considered a virtue is also an important matter since a flourishing life is obviously desirable. That being said, it should be kept in mind that dispositions which contribute to a flourishing life may lead to flourishing under a certain range of circumstances without leading to flourishing under all circumstances. For instance, certain characteristics may contribute to flourishing *only* when possessed by a critical number of individuals within a social group. Further, characteristics may contribute to flourishing when possessed in clusters but not on their own. Thus, under certain circumstances, an individual may suffer despite, and even as a result of, expressing virtue. Likewise, an individual may not experience physical health despite expressing virtue.

Over the past several years, flourishing has received increased attention in psychological science, and a number of studies have identified psychological characteristics that correlate with psychological growth, psychological well-being and/or physical health. Dispositional resilience has been positively correlated with successful adaptation to life stress (Ong et al., 2006) and with the well-being of widows (O'Rourke, 2004; Rossi et al., 2007). Additionally, dispositional mindfulness has been positively associated with both psychological well-being and physical health (e.g., Bernstein et al., 2011; Baer et al., 2012; Bowlin and Baer, 2012; Tamagawa et al., 2013); and individual differences in dispositional mindfulness predict psychological health (e.g., Baer, 2003; Baer et al., 2004). Other dispositions positively associated with psychological and/or physical health, include gratitude (e.g., Wood et al., 2010; Emmons and Mishra, 2011), optimism (e.g., Scheier and Carver, 1987; Scheier et al., 1989, 2001; Engberg et al., 2013; Carver and Scheier, 2014; He et al., 2014), self-efficacy (e.g., Bandura, 2004; Luszczynska et al., 2005), compassion (e.g., MacBeth and Gumley, 2012), altruism (e.g., Brown et al., 2009), self-regulation (e.g., Nix et al., 1999; Wrosch et al., 2003; Deci and Ryan, 2008b; Simon and Durand-Bush, 2014), forgiving (e.g., Berry and Worthington, 2001; Farrow et al., 2001; Maltby and Day, 2001; Seybold and Hill, 2001; Lawler-Row, 2010), spirituality (e.g., Hill and Pargament, 2003; Miller and Thoresen, 2003; Kuo et al., 2014; Reutter and Bigatti, 2014), religiosity (e.g., Hummer et al., 1999; McCullough et al., 2000; Oman and Thoresen, 2005; Greenfield and Marks, 2007; Park, 2007), and wisdom (e.g., Webster and Deng, 2014).

Most of the studies associating dispositions with flourishing—including those mentioned above—rely on surveys to measure the disposition in question. They examine associations between the possession of these dispositions and some measure, or correlate, of flourishing. This approach, however, is not ideal for measuring, or then studying, dispositions for several reasons.

First, surveys assessing dispositions do not involve measuring the *expression* of dispositions within the context of an individual's daily life; or allow directly associating this expression with flourishing. Rather, as we noted at the beginning of the previous section, surveys ask for generalized retrospective judgments removed from a person's daily context, which are susceptible to recall biases.

Second, as they do not involve repeated, cross-situational sampling, surveys assessing dispositions cannot effectively measure *intrasubject variability* in the expression of a disposition. As a result, this approach does not provide an effective means of measuring dispositional stability or robustness, which, as we have already seen, is paramount to the psychology study of virtues.

In contrast, EMA allows: (1) the detection of dispositional expression, and its correlates, within the context of daily life using multiple measures; and (2) the measurement of dispositional stability and robustness through repeated cross-situational sampling. EMA, thus, provides (3) a more thorough and direct means of examining the relationship between dispositions and flourishing than traditional approaches that rely on surveys. We will discuss points (1)–(3) in sequential order.

(1) Through the incorporation of environmental, activity, and physiological sensors, app-based EMA opens up various ways of detecting:

- the expression of virtues;
- correlates of having or expressing these dispositions; and
- the relevant features of the situations in which they are expressed.

Virtues can be expressed by psychological states (e.g., emotional states, motivational states) as well as by what people think and do (e.g., Bartlett and DeSteno, 2006; DeSteno et al., 2010). Thus, EMA can be used to detect virtue expression by asking people questions pertaining to their recent or current psychological states, experiences, thought life and/or behavior (for e.g., see studies listed in Table 2) as well as by administering brief psychological tests (cf. Schlicht et al., 2013). An EMA app can prompt individuals to respond to questions, or take brief tests, repeatedly at various moments, and across various situations, throughout the day. As a result, rather than asking people to make generalized retrospective judgments (e.g., “In the past month, I have… ”), an individual can be asked about their current or recent states, experiences or conduct (e.g., “Over the past hour, I have… ”). And survey-style instruments assessing virtues might be adapted so that, rather than asking for generalized judgments, they ask for reports concerning the present or recent past (cf. Fleeson, 2001).

For example, Hofmann et al. (2014) recently used EMA to repeatedly prompt people at random times over a 3-day period to report moral and immoral behavior over the previous hour. This allowed for the detection of patterns in moral behavior (e.g., social contagion, moral licensing) and awareness (e.g., a relative tendency to note others' immoral rather than moral behavior). More recently, Bleidorn and Denissen (2015) took adjectives associated with, and listed in, Peterson and Seligman's (2004) six *Virtues in Action* classifications (see Table 4) that could be meaningfully inserted into the following sentence: “I behaved particularly… during the last hour.” They, then, used app-based EMA to deliver these sentences to participants up to six times a day over a 10-day period in order to have them rate their behavior in the past hour. Amongst their participants—working mothers and fathers—they found that an individual's average virtue rating, the degree of variability in their rating, and the way an individual typically responded in certain contexts were relatively stable.

In addition to delivering questions, smartphones can be used to randomly capture conversations or other activities (e.g., Mehl et al., 2012). Further, prompts can be given immediately after these recordings asking individuals to report their states, experiences or thoughts; and/or asking them to upload a picture or video of their surroundings. This allows the association of life events and situations with momentary states, experiences and/or responses that would, otherwise, be forgotten once individuals are more temporally and spatially removed from the event or situation. It also allows the capture of contextual details that an individual would, otherwise, be unaware of or forget.

Activity sensors (e.g., Fitbit, Polo tech, Apple Watch; Moviesens) can also be used to record any physical and physiological activities correlated with an individual's momentary responses, states and/or experiences (e.g., D'Antono et al., 2001; Schwerdtfeger and Scheel, 2012; Bossman et al., 2013; von Haaren et al., 2013; Demarble et al., 2014; Dunton et al., 2014). Participants might, further, be prompted to take saliva samples in order to examine potential biochemical correlates (e.g., cortisol, oxytocin or progesterone levels) of dispositional expression (e.g., Brown et al., 2008; Entringer et al., 2011; Koven and Max, 2014). Or they might take a pharmacological agent (e.g., tryptophan, an anti-anxiolytic, an anti-depressant) while participating in an EMA study targeting dispositional expression (cf. Moskowitz et al., 2001; Moskowitz and Young, 2006).

**Figure 1:**
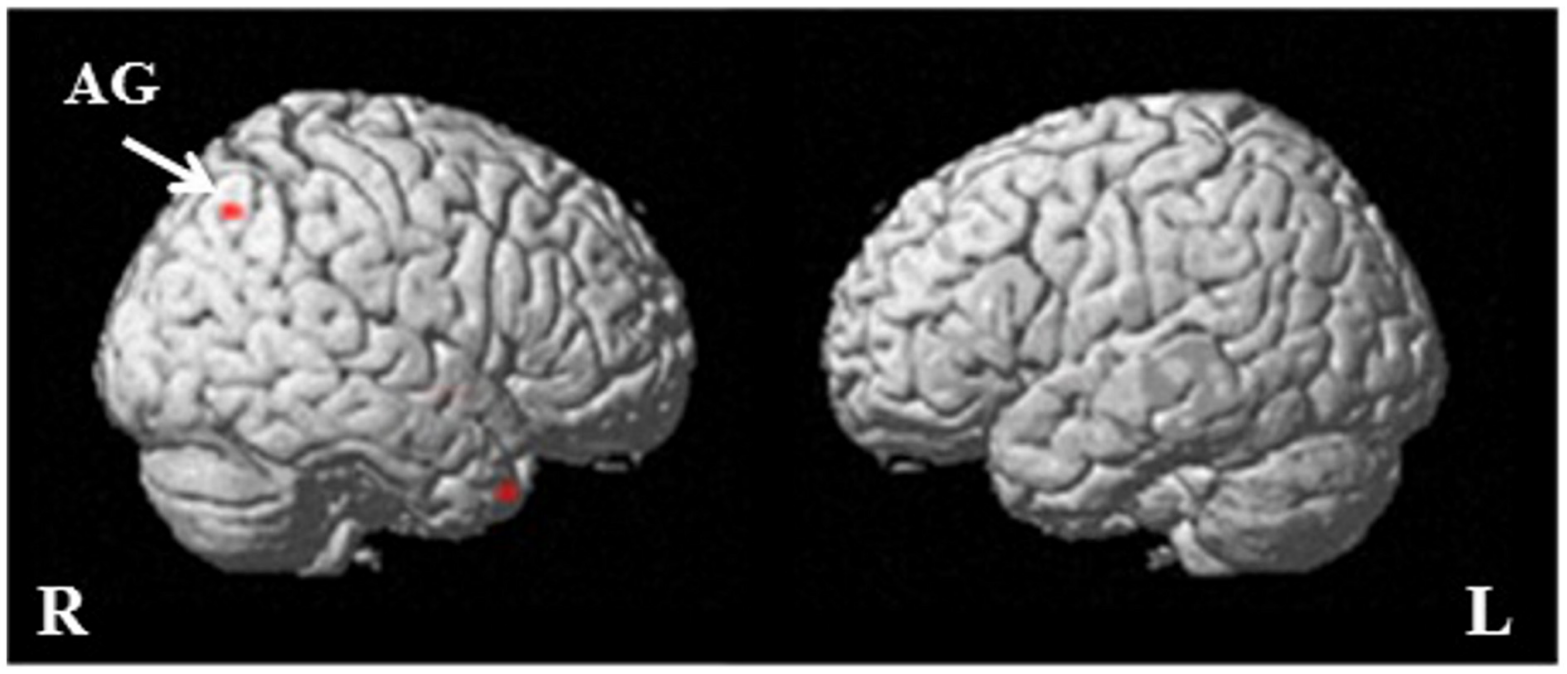
**Neural differences between forgiving and not forgiving as measured by fMRI**. The red shows where increased activity was observed when participants forgave in comparison to when they did not (taken from Strang et al., 2014). AG, angular gyrus; R, right; L, left.

In the near future, app-based EMA will also allow the isolation of neurophysiological correlates of having and/or expressing certain dispositions within certain situations. For example, mobile electroencephalography (EEG) caps (e.g., Eegosports) could be synced with an EMA app in order to record event-related potentials, or preparatory neural activities such as readiness-potentials (RPs); i.e., relative changes in the activity of the primary motor cortex and surrounding regions associated with preparedness to act (e.g., Freude and Ullsperger, 1987; Coles et al., 1988; Coles, 1989; Deecke et al., 1990; Shibasaki and Hallett, 2006; Ibanez et al., 2012; Nachev and Hacker, 2014). Through these means we might find that, when an individual has a certain disposition, certain preparatory activities occur under certain situations. Similarly, app-based EMA that incorporates mobile EEG might allow the detection of other neurophysiological correlates of expressing dispositions similar to those recently measured for forgiving using *f*MRI, where an increase in activity in the angular gyrus was associated with forgiving (see Figure 1).

(2) By allowing various ways of detecting (i)–(iii)—listed above—smartphone app-based EMA provides a vehicle for more direct and repeated measurement of disposition-relevant responses across various daily situations using an aggregate of measures. It, thus, provides a means of collecting cross-situational data to populate a frequency distribution of an individual's disposition-relevant responses organized by the degree to which each expresses the disposition in question. From this distribution, a mean score for an individual's dispositional expression and the variability of this expression can be calculated (Fleeson and Noftle, 2008). In this way, EMA provides a way of measuring the typical degree to which, and consistency with which, an individual expresses a disposition throughout the relevant situations of their daily life over a period of time. So it provides a means of directly measuring the *stability* and *robustness* of a disposition, or virtue. In so doing, it provides a way for assessing not simply *whether* an individual has a virtue but the *degree* to which they have a virtue.

**Figure 2:**
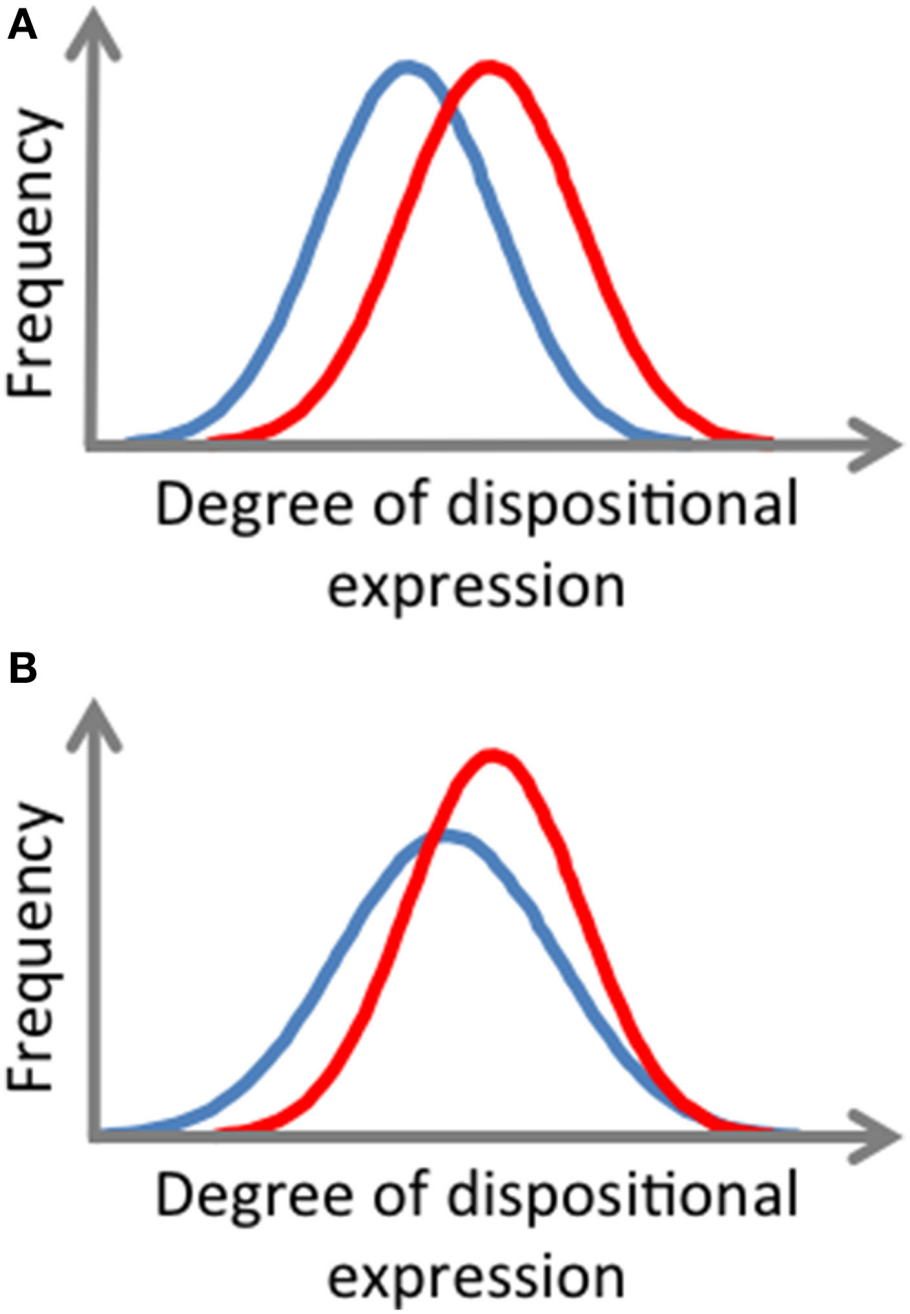
**Illustrative frequency distributions of momentary, cross-situational dispositional expressions displaying differences in degree of virtue possession. (A)** The red curve displays a distribution representing a higher typical degree of virtue expression (higher mean score) than the blue curve. **(B)** The red curve represents a higher typical degree of (higher mean score), and more consistent (less variable), virtue expression than the blue curve.

We should expect individuals who possess a certain virtue to *typically* express that virtue across a certain range of situations (Jayawickreme and Chemero, 2008). That is, given an Aristotelian conception, we should expect a virtue to be, to a certain degree, stable and robust. However, similar to the way the expression of other relatively stable and robust dispositions have been observed to vary (Ozer, 1986; McNiel and Fleeson, 2006; John et al., 2008; Donnellan and Lucas, 2009), some variability in the expression of a virtue should also be expected (see Miller, 2013). Nevertheless, the stronger, or more formed, a virtue, the more consistency there will be in its expression across relevant situations. This is because the stronger a virtue, the more frequently it is expressed in demanding situations, and despite interfering factors (Miller, 2013). So, after repeated cross-situational sampling of virtue-relevant responses, the degree to which an individual has a virtue can be measured as a function of the individual's mean score for its expression and the variability with which they express the virtue across relevant situations (cf. Fleeson, 2001; see Figure 2).

Thus, to recapitulate, EMA provides a way of repeatedly measuring virtue-relevant responses across various situations. This allows the measurement of the degree to which a person has a virtue along two dimensions: the degree to which they typically express the virtue and the consistency with which they express the virtue. These observations are in keeping with observations made concerning distributions of responses relevant to the expression of dispositions more generally (e.g., Mischel, 1968; Epstein, 1979, 1983; Fleeson, 2001; Fleeson and Noftle, 2008).

(3) As observed above, EMA provides a means of repeatedly and more directly assessing dispositional expression across an individual's daily situations. It, thus, provides a vehicle for not only assessing whether an individual has a virtue, but also the degree to which they typically express a virtue and the consistency with which they express a virtue. And since the degree to which an individual typically expresses a virtue and the consistency with which they express the virtue have implications for flourishing, EMA provides a thorough means of examining the relationship between virtues and flourishing.

To illustrate, EMA opens up a way of measuring *the degree to which an individual is typically grateful* and *the consistency with which an individual is grateful*. And both should be expected to promote flourishing given gratitude is a virtue. Thus, by allowing a direct assessment of both, EMA provides a thorough and direct means of examining the relationship between dispositional gratitude and flourishing. Further, since EMA allows the detection of dispositional expression within daily life, it also provides a good means of examining what may mediate relationships between dispositions, like gratitude, and flourishing as well as the *interconnectedness* of virtues (e.g., whether developing dispositional gratitude might directly correlate with developing others).

Before continuing we should mention that there are several limitations associated with EMA. Asking participants to repeatedly respond to prompts and questions over time, and within daily life, places a high demand on participants thereby increasing the likelihood of participant dropout and decreasing response rates (e.g., Shiffman et al., 2008). To compensate, participants need greater incentive than with traditional surveys. Also, there are issues regarding the invasion of privacy, which must be carefully addressed (Trull, 2015). Further, under certain conditions, EMA has been shown to result in reactivity (cf. Shiffman et al., 2008).

Having examined the advantages and limitations of using EMA—and specifically app-based EMA—to study virtues, we will now examine recent developments in EMI and the possibilities they open up for promoting dispositional development.

## EMI, positive change, and smartphone apps

Clinicians and therapists have often sought ways to improve the impact of therapy between sessions and the efficacy of interventions (Heron and Smyth, 2010). With this aim, over the past several years, researchers have been exploring the use of mobile devices to intervene and interact with clients within the context and moments of their daily life. This form of intervention, called ecological momentary intervention (EMI), has—similar to EMA—been implemented using PDAs, phone calls, text messages and, most recently, smartphone apps. EMI has developed largely as an extension of interventions involving computer- and internet-based cognitive-behavioral therapy (CBT; e.g., Butler et al., 2006; Andersson and Cuijper, 2009; Moore et al., 2011; Spence et al., 2011).

Self-monitoring has long been known to, under certain conditions, raise self-awareness and promote positive behavioral development (e.g., Harris and Lahey, 1982; Korotitsch and Nelson-Gray, 1999; Shapiro and Cole, 1999; Shiffman et al., 2008; Cohen et al., 2013; Maas et al., 2013). Specifically, it has been theorized that being asked questions about one's momentary states, experiences, behaviors and/or thoughts close to the time and context of their occurrence may help one become more mindful of their occurrence thereby providing opportunity for change (cf. Goodwin et al., 2008)^5^. Recent evidence suggests that EMI may be particularly effective for self-monitoring and raising self-awareness (Robinson et al., 2013; Runyan et al., 2013). For instance, in a recent study, our lab used an EMA/I app (iHabit) to ask undergraduate freshman how they were spending their time at various points throughout the day for three separate weeks during a semester (Runyan et al., 2013). Compared to controls, at the end of the study freshman using the app reported wasting nearly twice as much time throughout the semester. Further, amongst those using the app—but not amongst controls—this self-report predicted semester GPA comparable to the best single predictors of first semester GPA (i.e., high-school GPA and ACT score). The implication is that using the app promoted self-awareness concerning time-management.

In addition to prompting self-monitoring, EMIs have been used to:

- motivate (Rodgers et al., 2005; Franklin et al., 2006);
- encourage engagement in practices or the use of previously learned skills (e.g., DBT coach, Rizvi et al., 2011);
- aid in the development of new skills (Villani et al., 2012; Bless et al., 2014);
- notify (Dulin et al., 2014) or distract (Rodgers et al., 2005) individuals when they are at risk of engaging in addictive behavior; and
- provide individuals with personalized summary data (Hurling et al., 2007; Bless et al., 2014).

Though most EMI studies using smartphone apps are in developmental stages (e.g., Pramana et al., 2014; Wenze et al., 2014), there is some initial evidence from a number of health, clinical, and therapeutic domains that, in certain forms, EMI may promote positive dispositional development (e.g., Heron and Smyth, 2010; Cohn et al., 2011; Donker et al., 2013; see Table 5).

**Table 5:** **Peer-reviewed studies reporting effective smartphone/iPod EMIs**.

**Study**	**Intervention target**	**Sensors**	**Tailoring**	**App-based**	**EMI frequency**	**EMI duration**	**Immediate Post-intervention effects**	**Long-term effects**
Obermayer et al., 2004	Smoking cessation		x		Variant; At least 2 per day; Combined with website	6 weeks	x^a^	
Rodgers et al., 2005	Smoking cessation		x		5 per day for 6 weeks; 3 per week thereafter	6 months	x	x
Franklin et al., 2006	Type 1 diabetes management		x		1 daily and 1 weekly message	12 months	x	
Kim and Jeong, 2007	Type 2 diabetes management		x		Weekly	6 months	x	
Joo and Kim, 2007	Weight management behavior				Weekly	12 weeks	x	
Hurling et al., 2007	Physical activity	x	x		Tailored; Combined with website and email	4 months	x	
Weitzel et al., 2007	Negative consequences of drinking		x		Daily surveys and messages	2 weeks	x	
Yoon and Kim, 2008	Type 2 diabetes management		x		Weekly	12 months	x	
Brendryen et al., 2008	Smoking cessation				Between 1 and 3 messages every 2 weeks; Combined with website, interview and email	12 months	x	x
Atienza et al., 2008	Dietary intake		x		2 per day	8 weeks	x	
King et al., 2008	Physical activity		x		2 daily assessments; 1 daily & weekly tailored	8 weeks	x	
Patrick et al., 2009	Weight loss		x		Variant; Typically 1–5 per day	4 months	x	
Rizvi et al., 2011	Borderline personality disorder and substance use disorder			x	On average 15 times total	10–14 days	x	
Burns et al., 2011	Major depressive disorder	x		x	Tailored	8 weeks	x^a^	
Pop-Eleches et al., 2011	Antiretroviral therapy adherence				Weekly	48 weeks		x
Granholm et al., 2012	Schizophrenia-related medication adherence, socialization and hallucinations				12 per day; Combined with initial training	12 weeks	x	
Villani et al., 2012	Stress			x	8 videos	4 weeks	x	
Kauer et al., 2012	Emotional self-awareness and depressive symptoms			x	Approximately 2 per day (self-initiated)	2–4 weeks		x
Watts et al., 2013	Depression		x		6 sessions; Combined with weekly assignments and email	n/a	x	x
Robinson et al., 2013	Eating behaviors and weight management		x	x	2.7 episodes on average (user-initiated)	27.5 days on average	x^b^	
Carta et al., 2013	Parenting strategies and child engagement				2 per day; Combined with training home visits and weekly phone calls	Not specified	x	x
King et al., 2013	Motivation and physical activity	x	x	x	Live wallpaper and 1 end of day intervention; Combined with user-initiated features, texts and weekly alerts if goals weren't met	8 weeks	x	
Wayne and Ritvo, 2014	Self-management of type 2 diabetes	x		x	User-initiated; Combined with phone calls and in person meetings	6 months	x	
Enock et al., 2014	Social anxiety		x		3 per day	4 weeks	x	
Bless et al., 2014	Auditory attention		x	x	2 per day	21 days	x	
Ben-Zeev et al., 2014	Schizophrenia			x	5.2 per day on average	1 month	x	
Bond et al., 2014	Sedentary time reduction	x	x	x	Dependent on activity levels; Combined with education session	3 weeks	x	
Dulin et al., 2014	Treatment of alcohol use disorders	x	x	x	Variant; Location-based	6 weeks	x	
Lane et al., 2014	Sleep, physical activity, social interaction	x	x	x	Constantly accessible summary data	19 days	x^c^	
Maddison et al., 2014	Self-efficacy and exercise				Variant; First 12-weeks more intense; Combined with website	2 months	x	
Mhurchu et al., 2014	Weight management behavior	x	x		Ave. 2 motivational texts per day; 2 self-monitoring texts per week; Combined with hard-copy “toolkit” and website	8 weeks; 4 week maintenance		x^d^
Kramer et al., 2014	Depression		x		10 per day for 3 consecutive days per week; Weekly face-to-face feedback sessions	6 weeks	x	x
Cranwell et al., 2014	Self-control				3 per day	4 weeks	X	
Macias et al., 2015	Psychiatric and physical well-being	x		X	1 semiweekly, 1 weekly and 1 end of study assessment; Combined with texts, digital readings and videos	4 weeks	X	

*These studies were located by searching Pubmed.com, and publications from peer-reviewed journals listed in Scholar.google.com, up to November 27th, 2014 (search terms: “ecological momentary intervention” and “smartphone”)*.

a *Did not use controls*.

b *Half of participants lost weight. However, there was no control group as this was an intervention feasibility study*.

c *These results are from a small pilot, feasibility study*.

d *A pre/post decrease in weight and body mass index (BMI) was observed but there was no control as this was mainly a feasibility study*.

For example, in a smoking cessation study, text messages were sent to participants about health practices. Participants were also sent motivational stories or distraction topics (sports, travel, general interest, etc.) during times they were likely to smoke (Rodgers et al., 2005). Nearly 1000 messages were designed for this study and were sent to individual participants based on factors such as smoking history and preferences. The intervention was largely successful. In comparison to controls, twice as many people in the EMI group reported that they had quit smoking after 6 weeks.

**Figure 3:**
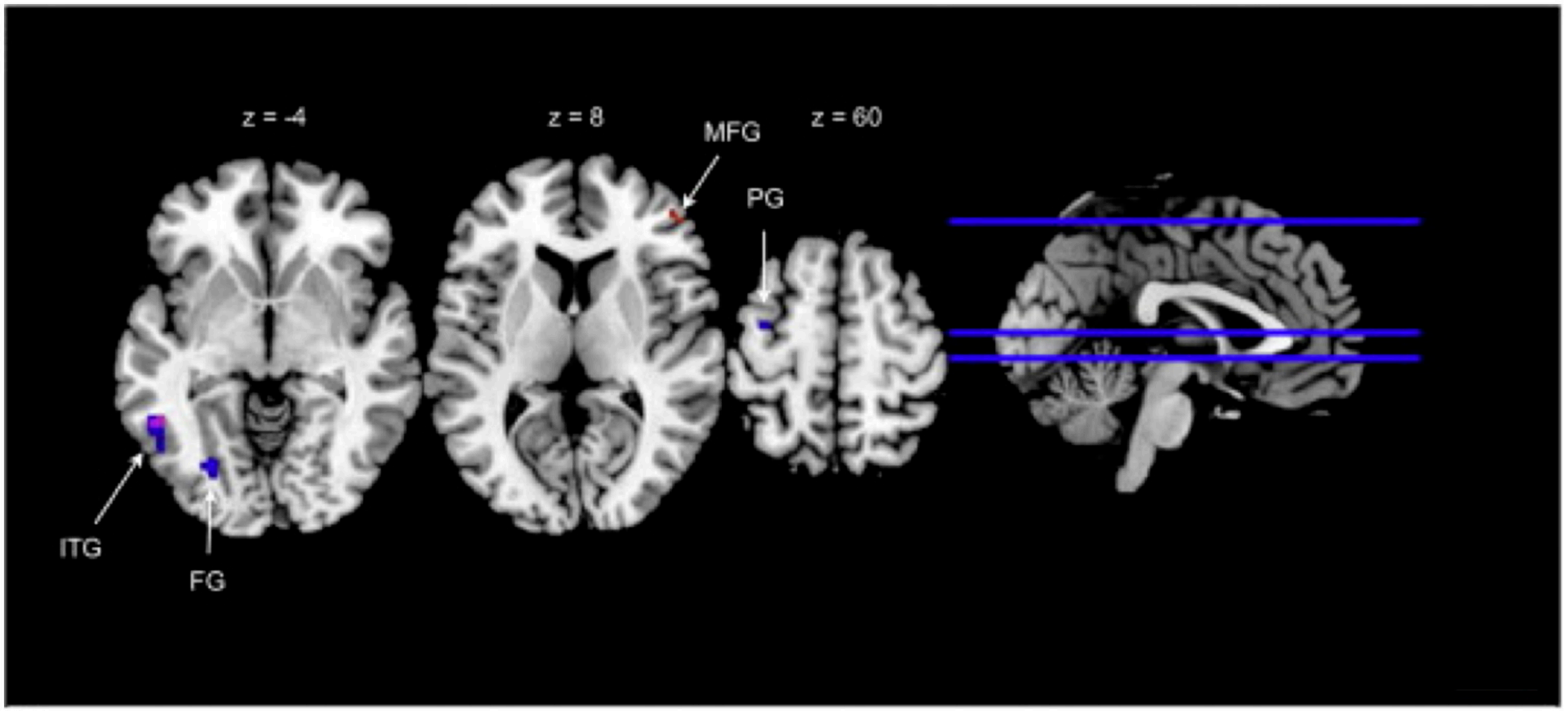
**Neural differences between those trained in an auditory attention task and controls as measured by fMRI**. Brain regions displaying significant decreases at points during a selective auditory attention task amongst trained participants (taken and adapted from Bless et al., 2014). Z, horizontal plane coordinate; ITG, inferior temporal gyrus; FG, fusiform gyrus; PG, precentral gyrus; MFG, middle frontal gyrus; red, forced-left response conditions; blue, forced-right response conditions; purple, overlap.

In another recent EMI study, participants performed a cognitive task to improve auditory attention on an iPod touch twice a day for 3 weeks (Bless et al., 2014). This experimental group, unlike the control group, showed improved auditory attention and evidence of functional neural plasticity in regions associated with auditory processing (left posterior temporal gyrus) and executive function (right middle frontal gyrus) during an auditory attention task as measured using fMRI (see Figure 3).

As we noted earlier, an important part of the psychological study of virtue is the examination of whether stable and robust dispositions can be developed. And though most app-based EMIs are in various exploratory stages, as we will discuss next, they hold promise for incorporating interventions that promote positive dispositional development into the daily activities of a large non-clinical, non-therapeutic population. In initial studies, effects during and immediately following EMI have been documented more than long-term effects (see Table 5). However, smartphone app-based systems that couple EMA and EMI provide an unprecedented means of testing whether EMI can help promote dispositional, including virtue, development.

## Virtues and app-based EMI

Little is directly known about the efficacy of EMI approaches to virtue development. In this section we, thus, discuss reasons for thinking such approaches have promise. In particular, we point out how app-based EMI offers a versatile, multifaceted and interactive way of promoting training, mindfulness, self-awareness, motivation and environmental awareness within the *context* of everyday life. We, then, outline parameters that may influence the effectiveness of EMI and an approach to optimizing EMIs for virtue development.

In addition to being practical due to the widespread use of smartphones, app-based EMI may be a particularly efficacious approach to promoting virtue development since it provides a versatile and multifaceted means of interacting with individual's within their everyday context. Developing a disposition, such as a virtue, is a learning process through which a behavior or response becomes a stable and robust habitual response or automatic response (e.g., Wood and Neal, 2007; Gawronski and Cesario, 2013). Learning context is important for this process.

There has been extensive animal research on the importance of learning context for response learning. First, animal studies reveal that learning contexts can function as “occasion-setters” such that, while they do not elicit a learned response themselves, they influence an animal's learned response to another stimulus thereby “setting the occasion” for this response (cf. Schmajuk and Holland, 1998; Bouton, 2010). Second, after learning a new response, animals often revert back to previous responses within contexts that vary from the context in which the new response was learned (cf. Bouton and Bolles, 1979; Peck and Bouton, 1990). Third, animals can be conditioned in one context (context A) and counterconditioned—or conditioned to give an opposing response—in another (context B), and continue to show the initial, conditioned response in context A and the opposing, counterconditioned response in context B (cf. Bouton and Bolles, 1979; Bouton and Peck, 1989; Merchant et al., 2013). Fourth, it has been observed that, under certain training conditions, a learned contextual response can persist even though memory for a conditioned stimulus (CS)/unconditioned stimulus (US) association has been impaired through localized inhibition of neurobiochemical processes crucial for the formation of long-term memory (i.e., memory persisting at least 48 h) for CS/US association (Runyan et al., 2004).

Extending from these animal studies, human studies have revealed that context is similarly important in the learning of habitual responses and automatic responses (e.g., Rydell and Gawronski, 2009; Wood and Neal, 2009; Gawronski and Cesario, 2013). Additionally, there is evidence that contextual cues can influence preparatory neural states associated with a certain response and the likelihood an individual will respond in that way in certain contexts (e.g., Deiber et al., 1996; Thoenissen et al., 2002; Toni et al., 2002; Praamstra et al., 2009; Moisa et al., 2012). In particular, addiction studies have shown that recovering individual's are more likely to relapse within contexts associated with the addictive behavior (e.g., Crombag et al., 2008); and this has been associated with plasticity in specific brain regions, including the lateral hypothalamus (e.g., Marchant et al., 2009, 2014). Further, there is evidence indicating that the tendency to give a habitual response becomes more *stable* and *robust* with the repetition of the response in various contexts (e.g., Bouton, 2000; Neal et al., 2006, 2011). Taken together, these observations provide evidence that, by promoting the development of habitual responses or automatic responses within an individual's daily context, EMIs aimed at virtue development may be particularly effective. Given this, EMA data coupled with GPS data could be used to trigger app-based EMIs in particular spatiotemporal locations to promote the learning of habitual or automatic responses in new, or various, contexts; especially in contexts where an individual finds change difficult (e.g., Watkins et al., 2014).

**Figure 4:**
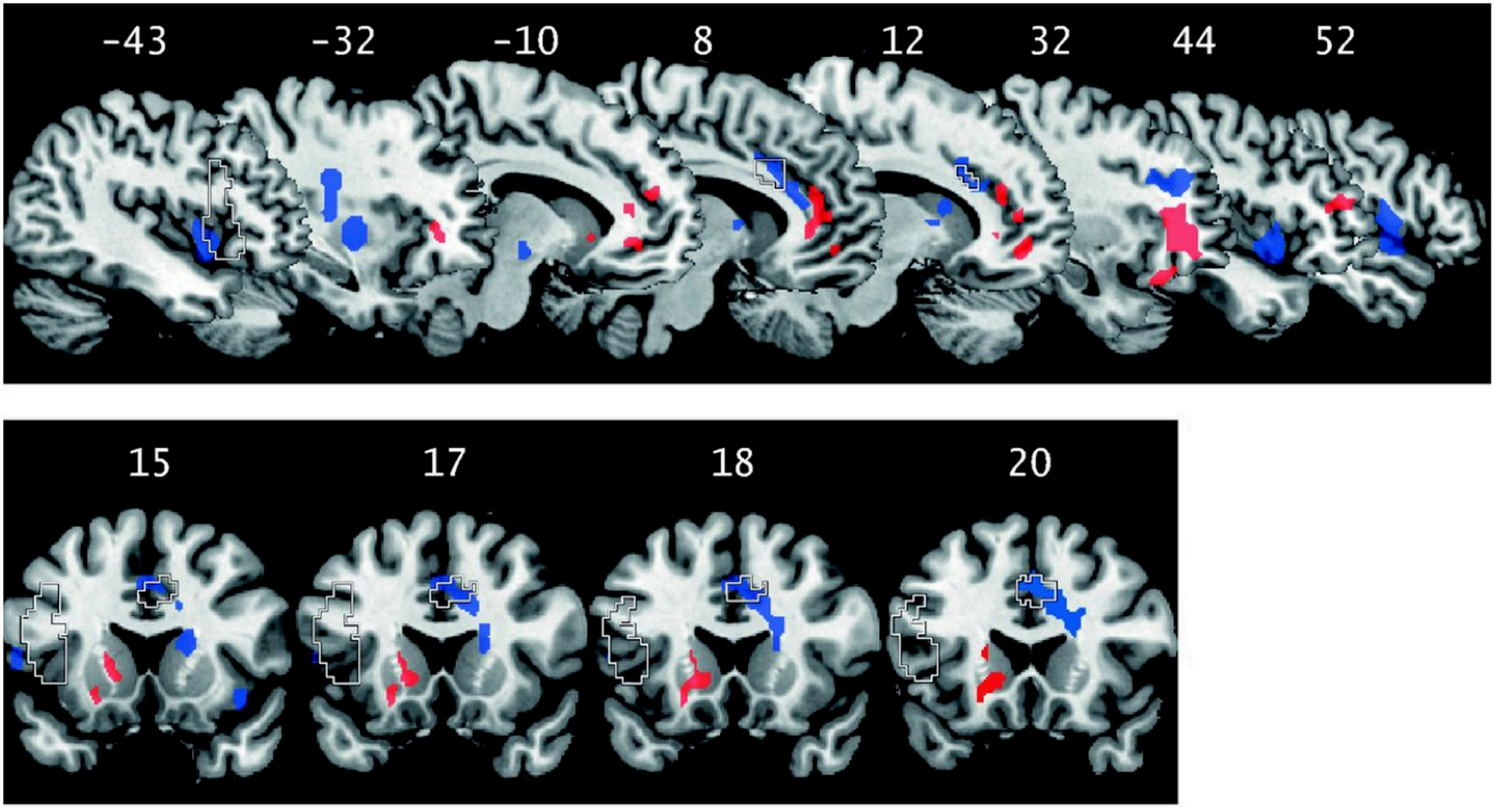
**Functional neural changes corresponding with empathy training (red) and compassion training (blue) in comparison to memory control as measured by fMRI (adapted from Klimecki et al., 2014)**. Dashed lines represent regions associated with empathy for pain in a recent meta-analysis by Lamm et al. (2011). Numbers on the top row are the sagittal plane coordinates. Numbers on the bottom row are the coronal plane coordinates.

One way that EMI might be effective in promoting virtue development is by prompting individuals to engage in practices, or in training, aimed at developing a particular virtue (cf. Magidson et al., 2014). Working memory training has been shown to improve cognitive abilities and to result in neural plasticity (e.g., Klingberg, 2010). There is some suggestion that practicing gratitude increases dispositional gratitude (Emmons and McCullough, 2003; Seligman et al., 2005). Additionally, self-regulation exercises have been shown to improve self-regulation (Baumeister et al., 2006; Cranwell et al., 2014). Further, in a recent study, participants were given empathy and compassion training (Klimecki et al., 2014; cf. Klimecki et al., 2012). After empathy training, participants experienced increased negative affect associated with increased activity in the anterior insula and the anterior midcingulate cortex (two regions previously associated with empathy for pain) in response to watching videos depicting human suffering. After compassion training, these same individuals also experienced increased positive affect associated with increased activity in the ventral striatum, pregenual anterior cingulate cortex and medial orbitofrontal cortex (see Figure 4). The same effects were not found in controls who underwent memory training. The implication is that training can result in increased empathy and compassion as measured by affective responses and associated brain activity states.

App-based EMI may be a particularly effective way of administering dispositional training of the kind mentioned above since smartphone app technology can support multifaceted, interactive and progressive training within various contexts throughout an individual's daily routine. For instance, training might involve prompting individuals to engage in app-delivered activities or exercises, interact with app-based games, or with videos or pictures, throughout the day. And training might get more demanding over time.

Another way EMI might promote virtue development is by specifically targeting mindfulness. According to the predominating definition in psychology, mindfulness is purposeful, non-evaluative awareness of one's present experiences and mental states moment-to-moment (Kabat-Zinn, 2003; but see Brown and Ryan, 2003; Jankowski and Holas, 2014). Not only is mindfulness thought by some to be a virtue—and there is evidence that dispositional mindfulness does promote mental health and resilience (e.g., Brown et al., 2007)—there is some suggestion that mindfulness may help promote the development of other virtues and, thus, provide a case where virtues are interconnected. It is theorized that mindfulness promotes positive change by promoting awareness of one's immediate experiences and states from a somewhat detached state, which may, in turn, promote self-awareness and self-regulation (Shapiro et al., 2006; Jankowski and Holas, 2014).

EMA observations support the theory that mindfulness promotes self-regulation (e.g., Brown and Ryan, 2003). Additionally, mindfulness training (involving attentional fixation on and nonjudgmental awareness of moment-to-moment experiences) can improve working memory and attention (Tang et al., 2007; Lutz et al., 2008; Jha et al., 2010; MacLean et al., 2010). These improvements have been associated with increased activity in the left dorsolateral prefrontal and dorsal anterior cingulate cortex (Allen et al., 2012). Mindfulness has also been observed to reduce interference from emotionally salient distractors (Ortner et al., 2007), which has been associated with increased activity in the medial prefrontal cortex and right anterior insula (Allen et al., 2012).

App-based EMI approaches to virtue development might prompt and remind individuals to engage in dynamic and progressive mindfulness exercises within the context of daily life. There are a number of mindfulness apps currently available (Plaza et al., 2013). However, at present there has been little work on the effectiveness of app-based EMIs aimed at promoting mindfulness (but see Chittaro and Vianello, 2014). Further, as we will see toward the end of this section, there are a number of parameters that are likely to influence whether EMIs are efficacious.

In addition to prompting various practices, trainings or exercises within everyday contexts, effective app-based EMIs for virtue development might be designed by incorporating effective components of computer- and internet-based therapeutic interventions (cf. Kaltenthaler et al., 2006; Barak et al., 2008; Tillfors et al., 2008; Andersson, 2009; Bergström et al., 2010; Newman et al., 2011; Andersson et al., 2013; Musiat and Tarrier, 2014). Web-based cognitive-behavioral therapy and guided self-help interventions have been observed to be efficacious in treating eating disorders (Hötzel et al., 2013), insomnia (Holmqvist et al., 2014), suicidal ideation, depressive symptoms, anxiety (van Straten et al., 2008; March et al., 2009; Cuijpers et al., 2013), posttraumatic stress disorder (Knaevelsrud and Maercker, 2007) and physical inactivity (van Stralen et al., 2009a,b, 2010, 2011). In multiple cases, these interventions have been observed to facilitate long-term change (e.g., Knaevelsrud and Maercker, 2007; Litz et al., 2007; March et al., 2009; Ruwaard et al., 2010; van Stralen et al., 2011; Cuijpers et al., 2013; Lappalainen et al., 2014).

In these computer- and internet-based approaches:

1. self-monitoring and self-awareness (e.g., Litz et al., 2007; Morris et al., 2010),
2. self-efficacy and motivation (Litz et al., 2007; Turner et al., 2007; Carlbring and Smit, 2008; Warmerdam et al., 2010; Hötzel et al., 2013), and
3. environmental awareness (van Stralen et al., 2009a)

are among the strongest mediators of enduring change. And, as we will discuss in turn below, app-based EMI might be used to effectively promote each.

(1) As mentioned in the previous section, research from our own lab shows that using an EMA/I app to ask individuals questions about their responses at random times throughout their daily activities can raise self-awareness (Runyan et al., 2013). Additionally, app-based EMIs might allow individuals to monitor summary data generated from their responses. Further, by incorporating sensors, app-based EMI provides a means of increasing self-awareness by increasing a person's ability to self-monitor. App-based EMIs that interface with mobile EEG (cf. Curran and Stokes, 2003), physical activity sensors and/or other physiological activity sensors (e.g., sensors for muscle tension, temperature, galvanic skin response, blood pressure or heart-rate; see Sutarto et al., 2010; Schwerdtfeger and Scheel, 2012; Bossman et al., 2013; von Haaren et al., 2013; Demarble et al., 2014; Dunton et al., 2014) could provide biofeedback allowing an individual to self-monitor to an extent otherwise impossible within close spatial and temporal proximity to a focal event or state (cf. Keedwell and Linden, 2013; Linden, 2014; Schoenberg and David, 2014). Feedback concerning preparatory neural activities, muscle tension, blood pressure and/or heart rate (e.g., Koval et al., 2013) within certain contexts might enable an individual to monitor patterns in their behaviors, thought life, states and/or experiences of which they would otherwise be unaware.

(2) There is indication that self-monitoring promotes self-regulation and positive behavioral development when individuals have the opportunity and motivation to change (cf. Bandura, 1991; Korotitsch and Nelson-Gray, 1999; Shiffman et al., 2008; Quinn et al., 2010). Therefore, app-based EMIs might effectively promote positive change not only by providing a means for self-monitoring but by also increasing motivation through:

- repeatedly asking individuals directed questions;
- prompting goal-setting;
- pushing motivational statements, videos or pictures and promoting positive self-talk to increase outcome expectancy (i.e., belief that one's efforts will lead to the achievement of a desirable end) and self-efficacy (i.e., belief in one's ability to achieve one's goal; Bandura, 1986, 1997);
- reminding individuals of their own intentions and values; and/or
- intermittently giving individuals progress and accomplishment reports (which has been shown to increase motivation and promote positive behavioral development; e.g., Korotitsch and Nelson-Gray, 1999; Kramer et al., 2014; Mhurchu et al., 2014).

Also, a social component to app-based EMI, where individuals can interact with others who are using the same EMI in order to develop the same virtue, might also increase motivation as well as self-efficacy (cf. Obermayer et al., 2004; Przeworski and Newman, 2004; Hurling et al., 2007; Brendryen et al., 2008; Cafazzo et al., 2012; King et al., 2013).

(3) With regard to increasing environmental awareness, app-based EMIs that incorporate GPS (e.g., Yüce et al., 2012; Hollett and Leander, 2013; Huang and Luo, 2014; Watkins et al., 2014), and utilize smartphone microphones and cameras, might be designed to notify individuals about aspects of their environment. In this way, app-based EMIs might be designed to raise awareness of contextual/situational triggers for responses that the individual desires to change as well as awareness of opportunities to respond in ways that are expressions of a virtue. App-based EMIs might also raise awareness of environmental resources and/or social support that may help an individual in their effort to change (e.g., van Stralen et al., 2009a; Mhurchu et al., 2014).

Whether EMI can effectively promote virtue development—and the optimal conditions for this development—remains to be directly and systematically tested. In particular, it remains to be seen whether EMIs, including app-based EMIs, can promote long-term dispositional development that persists following the termination of the intervention. Relevant parameters of EMI in need of testing are:

- duration of intervention;
- frequency of interactions;
- duration of each interaction;
- whether interactions occur at varied or fixed times;
- whether, and the degree to which, the intervention is intermittent or continuous over a period of time;
- degree of variation or repetition between interactions;
- how broadly or narrowly the intervention is focused (e.g., number of responses targeted, types of interactions provided);
- whether the intervention is individualized/tailored;
- whether the intervention progresses over time (e.g., involves scaffolding);
- whether the intervention involves social interaction; and
- the degree to which the intervention is engaging (e.g., interesting, meaningful and/or fun).

Given EMI can promote virtue development, optimal conditions are likely to depend somewhat on the virtue. Nevertheless, there are also likely to be some common optimal conditions. We hypothesize that these optimal conditions will, to a certain degree, track those for instrumental learning whereby intentionally modified responses in early stages of learning are cued by stimuli as expressions of a habitual response or automatic response in later stages (e.g., Dickinson et al., 1995; Schachtman and Reilly, 2011). In this case, EMIs that maintain the highest levels of engagement, motivation and awareness of opportunities to respond or otherwise behave in ways that are expressions of a virtue, over the longest period of time, are likely to be the most effective (e.g., Rescorla and Solomon, 1967; Sutherland and Mackintoch, 1971; Rescorla and Wagner, 1972; Bandura, 1977). This is likely to be accomplished by EMIs that:

1. focus on one virtue at a time (cf. Kamin, 1968; Rescorla and Wagner, 1972);
2. progress and involve scaffolding (cf. Salomon, 1993; Pea, 2004);
3. are tailored to the individual and incorporate the individual's judgments, values and aims (cf. Levey and Martin, 1975; Shanks and Dickinson, 1991; Kreuter et al., 2000a,b; Strecher et al., 2005);
4. involve social interaction amongst individuals with similar aims (cf. Bandura, 1977, 1986, 1997); and
5. interact with individuals at varied, rather than fixed, intervals and provide positive reinforcement for virtue development (e.g., make virtue development, and interaction with the intervention, varied, interesting, meaningful and/or fun).

Scheduling intermittent EMAs as an individual uses an EMI aimed at promoting virtue development provides a way of assessing and optimizing the efficacy of EMI in real-time (cf. Voogt et al., 2013). As we have already discussed, EMA can be used to assess virtue expression, and/or detect correlates of this expression. And a smartphone app can be used to administer both EMA and EMI.

## Conclusions

There seems to be conclusive reasons for thinking that some people possess virtues understood as relatively stable and robust psychological dispositions that contribute to a deeply fulfilling, well-lived life of growth (i.e., a flourishing life)—and situational studies have not presented reasons for thinking otherwise. Further, since knowledge of what contributes to flourishing is worth seeking, virtues are worth studying. As a result of the widespread use of smartphones and advancements in smartphone technology, app-based EMA provides a new means for examining the stability, robustness and interconnectedness of virtues, and the physiological (including neurophysiological) correlates of having and/or expressing virtues. In short, app-based EMA provides a wholistic approach to examining the extent to which virtues are possessed as well as the personal, physiological and biochemical characteristics of individuals who possess and express virtues, which promises to provide new insight. Additionally, app-based EMI provides a new and powerful vehicle for promoting the development of virtues where virtues should be expressed—within the context of our daily lives.

### Conflict of interest statement

The corresponding author is a co-creator of iHabit and LifeData systems and is a founding partner of LifeData, an LLC which creates mobile device-based ecological momentary assessment and intervention systems.

## Footnotes

^1^It should be noted that the Aristotelian tradition is a eudaimonic tradition in which happiness, or flourishing, is analyzed in terms of living a fulfilled and deeply satisfying life of growth through expressing virtues (Kenny, 2011, pp. xii–xiv). This tradition is in opposition to hedonic traditions in which happiness is analyzed in terms of affect or subjective well-being (cf. Deci and Ryan, 2008a). At the same time, accepting a eudaimonic analysis does not entail, or typically involve, thinking it can never be the case that a person who is exercising virtues is not flourishing; or that a flourishing life will not include subjective well-being or pleasure.

^2^Two points should be made here: (1) First, Allport, and many psychologists since, talk of traits (i.e., personality traits, character traits) rather than psychological dispositions; and, while, “trait” and “disposition” are commonly used interchangeably, traits are often thought of as a type of more stable disposition. Having said this, there are variations in the use of the term “disposition.” Sometimes, “disposition” and “trait” are both used to refer to any relatively stable and robust psychological characteristic, including deficiencies or the lack of certain psychological abilities (e.g., Eysenck and Eysenck, 1969; Watson and Clark, 1984; McCrae and Costa, 2003). Other times, often in philosophical psychology, a psychological disposition is understood as any psychological ability, some of which are not, in and of themselves, typically understood as a trait or aspect thereof (e.g., Annas, 2011; Anjum et al., 2013; Hyman, 2014). For our purposes here, it is not crucial to analyze various conceptions of psychological traits or dispositions, or how these conceptions overlap. It suffices to point out that, often, there is overlap in the use of “trait” and “disposition,” and, for our purposes here, a psychological disposition is a psychological ability. A virtue is, then, a subclass of psychological disposition or ability as outlined above (also see Mumford, 1998). (2) Second, according to Allport (1960), the study of virtues involves value judgments and, thus, is a study for moral philosophy rather than psychological science. As will become clear, we take a different view.

^3^Most smartphone app-based EMA systems work on either Apple or Android devices, which make up the majority of smartphones in use. Only two work with both: illumivu and LifeData.

^4^Psychological well-being is not to be confused with subjective well-being (for e.g. see Ryff and Singer, 2008; Ryff, 2013). See note 1.

^5^It should be noted that it has also been theorized that simply being aware of being observed, or of having one's behavior assessed, effects one's behavior—a phenomenon known as the Hawthorne effect (Solomon, 1949; Sommer, 1968; Parsons, 1974; Wickstrom and Bendix, 2000). However, recently, mixed evidence for the Hawthorne effect has lead to serious questions about the adequacy of this construct. Rather than there being a Hawthorne effect, evidence indicates that multiple conditions lead to various effects when one is aware of being assessed or of having one's behavior assessed (see Gale, 2004; McCambridge et al., 2014). Here, we focus on one such condition: increased self-awareness.
